# Effect of hyoscine-N-butyl bromide rectal suppository on labor progress in primigravid women: a randomized double-blind placebo-controlled clinical trial

**DOI:** 10.3325/cmj.2011.52.159

**Published:** 2011-04

**Authors:** Somayeh Makvandi, Mitra Tadayon, Mohammadreza Abbaspour

**Affiliations:** 1 Department of Midwifery, Faculty of Nursing and Midwifery, Ahvaz Azad University, Ahvaz, Iran; 2 Department of Midwifery, Faculty of Nursing and Midwifery, Ahvaz Jundishapur University of Medical Sciences, Ahvaz, Iran; 3 Nanotechnology Research Center and School of Pharmacy, Ahvaz Jundishapur University of Medical Sciences, Ahvaz, Iran

## Abstract

**Aim:**

To determine the effects of hyoscine-N-butyl bromide (HBB) rectal suppository on labor progress in primigravid women.

**Methods:**

A randomized double-blind placebo-controlled clinical trial was carried out on 130 primigravid women admitted for spontaneous labor. The women were recruited based on the inclusion and exclusion criteria and randomized into the experimental (n = 65) and control group (n = 65). In the beginning of the active phase of labor, 20 mg of HBB rectal suppository was administered to the experimental group, while a placebo suppository was administered to the control group. Cervical dilatation and duration of active phase and second stage of labor were recorded.

**Results:**

The rate of cervical dilatation was 2.6 cm/h in the experimental and 1.5 cm/h in the control group (*P* < 0.001). The active phase and the second stage of labor were significantly shorter in the experimental group (*P* = 0.001 and *P* < 0.001, respectively). There was no significant difference between the two groups in the fetal heart rate, maternal pulse rate, blood pressure, and the APGAR score 1 and 5 minutes after birth.

**Conclusion:**

Use of HBB rectal suppository in the active management of labor can shorten both the active phase and second stage of labor without significant side-effects.

**Registration No:**

IRCT138804282204N1.

Active management of labor reduces the number of cesarean deliveries, the number of prolonged labors, and labor duration, without having any adverse effects on the mother or the fetus (1-5). Spasmolytics, such as hyoscine -N-butyl bromide (HBB), are commonly used during this process. Several studies, which included both primigravid and multigravid women, have shown that intravenous application of HBB (20-40 mg) during the active phase of labor increases cervical dilatation (6,7) and decreases duration of the first stage of labor (8-11). When given in the latent phase, during which the contractions are still not strong, HBB actually delays the labor progress by decreasing the intrauterine tension (8). Even though the rectal route is less invasive and would allow administration even by the patients themselves, effects of HBB given as a rectal suppository have been rarely studied. All of these studies involved both primigravid and multigravid women, which could be problematic in labor trials (9,10). In the study by Tiwari et al on 300 primigravid and multigravid women (11), 100 women received 20 mg of HBB intravenously, 100 women received 8 mg of valethemate bromide every 20 minutes at 4 cm dilatation, and 100 women received no drug. Significant shortening of the first stage of labor in primigravid women was noted in both experimental groups and it was more remarkable with HBB. In another study on the effect of intravenous HBB in the active phase of labor (9), the mean time of the first stage in the experimental group was 31.7% shorter than in the control group. Gupta et al performed a prospective randomized trial on 150 women in active labor, 50 of whom were given drotaverine, 50 HBB, and 50 no medication (12). The mean duration of the active phase and the mean rate of cervical dilation did not differ between the groups.

The present study assessed the effects and safety of a single dose (20 mg) of HBB administered as a rectal suppository during labor, since such administration of HBB has been demonstrated to be less invasive and simpler, even possible by the patient herself.

## Methods

The study design was approved by the institutional Ethics Committee of Ahvaz Jundishapur University of Medical Sciences and registered within the Iranian registry of clinical trials (*http://irct.ir*); IRCT No.: IRCT138804282204N1. The trial was conducted at the Sina hospital in Ahvaz, Iran, between July and October 2009.

A preliminary power analysis was carried out to calculate the sample size for the experimental and control groups using a formula recommended by previous studies: d = Δ/SD, where d is standardized difference, Δ is the smallest clinically significant difference, and SD is standard deviation of the test group. Duration of 60 minutes was considered as the smallest clinically significant difference, and the SD (68.9 minutes) was selected from the study performed by Sirohiwal et al (10). Also, a standardized difference of 0.87 was obtained using nomogram (13). The power analysis suggested that a sample of 70 women in each group would provide a power of 95%, at 5% significance.

### Participants

Women in labor were recruited from the antenatal clinic of our hospital after they had received information on the purpose and course of the study from the medical investigator and had provided the written consent during routine visits. The trial included primigravid women between 18-35 years of age with normal pregnancy, a singleton fetus at a gestational age of 37-42 weeks in a cephalic presentation, and in the beginning of spontaneous labor. Since a number of studies reported a clear association between maternal overweight and dysfunction of active phase of labor, specifically arrest of dilatation (14,15), we included only women with a body mass index lower than 25 before pregnancy. The beginning of the active phase of labor was defined as cervical dilatation of 3-4 cm in the presence of moderate uterine contractions. Moderate uterine contractions were defined as those during which the underlying fetal parts were not palpable, but fingers could still be indented in the abdominal wall. Exclusion criteria were maternal tachycardia (>100 bpm), antepartum hemorrhage, prolonged rupture of membranes, previous uterine scar, cephalopelvic disproportion, augmentation of labor using oxytocin, preeclampsia, heart disease, or any other serious medical conditions.

### Intervention

Suppositories containing 20 mg HBB (courtesy of Exir pharmaceuticals, Boroujerd, Iran) were prepared by a pharmaceutical technician who was not included the clinical trial, as were the placebo suppositories, which consisted of a Suppocire AM-15, a semi-synthetic fatty acid glyceride (courtesy of Gattefosse, Lyon, France). The suppositories were held refrigerated until the time of their usage. Using a block randomization method (block size = 4), random numbers were assigned to each package. The patients and the medical investigator were not familiar with the content of each package. The participants were given one of the rectal suppositories in the beginning of the active phase of labor.

### Outcome measures

Obstetric examinations and routine investigations were carried out in all cases by a single investigator. According to the standard procedure at our institute, vaginal examination was performed every 2 hours to assess the progress of labor. Routine amniotomy was performed for women who did not have a spontaneous rupture of membranes at the time when the presenting part of the fetus was fixed. A partogram was maintained throughout the labor. Duration of the active phase of labor, rate of cervical dilatation, and duration of the second stage of labor were recorded.

Neonatal APGAR scores were determined 1 and 5 minutes after birth. Fetal heart rate, as well as the patient’s pulse rate and blood pressure were recorded every half an hour. Following the RANZCOG intrapartum fetal surveillance clinical guidelines, fetal monitoring was done by intermittent auscultation using Doppler ultrasound at least every 15-30 minutes in the active phase of the first stage of labor and every 5 minutes in the second stage in the absence of active pushing, as well as after each contraction with active pushing in the second stage (16). In the presence of risk factors for fetal compromise such as abnormal auscultation, a continuous electronic fetal monitoring was used.

### Statistical analysis

Data were analyzed with SPSS, version 15.0 (2006, SPSS Inc., Chicago IL, USA). Absolute and relative frequencies of categorical variables and mean and standard deviation of continuous variables were calculated. Continuous variables were analyzed by *t* test, and χ^2^ test was used for categorical variables. When the χ^2^ test could not be applied for categorical variables, such as the first minute APGAR score, we used Fisher exact test.

## Results

**Figure 1:**
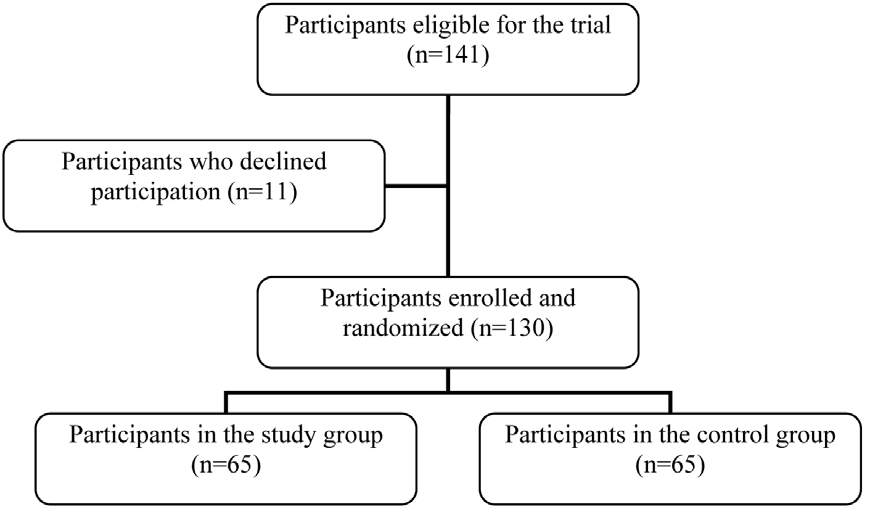
Flow of participants through the study on the effect of hyoscine-N-butyl bromide rectal suppository on labor progress in primigravid women

Participants’ flow through the study is shown in Figure 1. There was no significant difference in maternal characteristics between the experimental and control group (Table 1). The mean duration of the active phase of labor was 141.0 ± 81.7-minute in the experimental and 230.1 ± 169.6-minute in the control group (*P* = 0.001). The mean duration of the second stage of labor was 38.8 ± 24.3-minute in the experimental and 51.7 ± 23.8-minute in the control group (*P* < 0.001). The rate of cervical dilatation was 2.6 cm/h in the experimental and 1.5 cm/h in the control group (*P* < 0.001).

**Table 1:** Characteristics of women in the experimental group who received hyoscine-N-butyl bromide suppository and women in the control group who received placebo suppository

	Group (mean ± standard deviation or number, %)		
Characteristic	experimental (n = 65)	control (n = 65)	*P*
Age (year)	22.14 ± 3.72	22.38 ± 4.02	0.72
Body mass index before pregnancy (kg/m^2^)	23.34 ± 4.11	24.56 ± 5.78	0.52
Weight gain (kg)	13.14 ± 3.15	14.40 ± 3.84	0.48
Preterm rupture of membranes	25 (38.5)	25 (38.5)	1.00
Length of preterm rupture of membranes (h)	7.95 ± 1.89	8.41 ± 2.12	0.47
Cervical dilatation at intervention (cm)	3.83 ± 0.84	3.88 ± 0.71	0.74
Cervical effacement at intervention (%)*	64.15 ± 10.88	62.31 ± 9.81	0.31

*Thinning of the cervix as a percentage change from the baseline.

There was no significant difference in the rate of cesarean section between the groups (7.69% vs 9.23%; *P* > 0.05). The most common indication for a cesarean section was an arrest of dilatation, followed by thick meconium and placental abruption. There were no instrumental vaginal deliveries.

There was no significant difference in the fetal heart rate between the two groups (Table 2). Fetal heart rate variation in both groups was 7.7% (3.10% bradycardia and 4.60% tachycardia vs 4.60% bradycardia and 3.10% tachycardia; *P* > 0.05).

**Table 2:** Fetal and maternal outcomes (mean ± standard deviation) half an hour after giving hyoscine-N-butyl bromide suppository in the experimental group and placebo suppository in the control group

	Study group		
Outcome	experimental (n = 65)	control (n = 65)	*P*
fetal heart rate (per min)	140.87 ± 24.65	137.93 ± 26.61	0.51
maternal pulse rate (per min)	83.34 ± 10.56	86.65 ± 12.87	0.45
maternal systolic blood pressure (mmHg)	108.78 ± 12.34	110.09 ± 13.67	0.64

There was no significant difference between the groups in the maternal pulse rate and mean systolic blood pressures either half an hour after the intervention (Table 2) or at other time intervals (*P* > 0.05) (data not shown).

Because of the shortening effect of spontaneous preterm rupture of membranes upon the first stage of labor (17), we did not find any significant difference between the groups in the number and duration of ruptures.

The drug was well tolerated by all patients and no adverse effect was noted. Out of 130 neonates, 2 in the experimental group had an APGAR score of 4-6, while the rest had a score of 8-10 one minute after birth. The APGAR score 5 minutes after birth ranged from 8-10 in all 130 neonates.

## Discussion

The present study showed a significant decrease in labor duration when a 20 mg rectal suppository of HBB was used in the active phase of labor. Sirohiwal et al also found a significant difference in the duration of active phase of labor between the control and study group (368.1 ± 133.0 vs 123.9 ± 68.9-minute, respectively) (10), but the duration of second stage of labor was not affected. In our study, the second stage of labor was significantly shorter with HBB than with placebo. Although all women were encouraged to push at the beginning of the second stage of labor, the prolonged second stage could be related to the longer duration of the first stage of labor, which may have led to tiredness of the myometrium. On the other hand, Gupta et al found that the active phase duration and rate of cervical dilatation in the group that received HBB were not significantly different from the control group (12). Similar observations were also made by Mortazavi and Rakhshani, who reported a longer first stage of labor with intramuscular HBB (18). We believe our results were influenced by the local effect of the rectal suppository on the cervical region, although the exact mechanism of action is not established. It is possible that induced relaxation enables more effective myometrical contractions. Iravani and Bekhradinasab conducted a study using 20 mg intravenous HBB for active management of labor in 100 primigavid women with 100 primigavid women as control (7). They reported that 24% of fetuses in the experimental group and 10% of fetuses in the control group had heart rate variation (8% bradycardia and 16% tachycardia vs 2% bradycardia and 8% tachycardia). Our study showed no such difference, so it is possible that these differences could be an effect of intravenous application of HBB which, in comparison with rectal suppository, can rapidly alter the fetal heart beat.

Although our study was not sufficiently powered to assess adverse neonatal outcomes, the initial examination of each neonate, their APGAR score 1 and 5 minutes after the birth, showed no difference between the groups.

Since a primigravid woman can be fully dilated before she feels the pressure of giving birth, it is impossible to determine the exact time of the full cervical dilatation, although average time from administration of drug to delivery (total amount of active phase and second stage) was significantly shorter in the experimental group.

We believe our study showed that HBB rectal suppository was effective in shortening the duration of labor. Furthermore, the rectal administration of the drug is more convenient, absorption is faster, gastric irritation is avoided, and hepatic metabolism is partially bypassed (10). Further studies are necessary to fully evaluate the benefits of HBB, as well as its effect on women in induced labors or those with an active phase arrest or protraction disorders.
